# [6]-Shogaol Attenuates Oxaliplatin-Induced Allodynia through Serotonergic Receptors and GABA in the Spinal Cord in Mice

**DOI:** 10.3390/ph15060726

**Published:** 2022-06-08

**Authors:** Suyong Kim, Juan Gang, Ji-Hwan Lee, Hyejin Yang, Chunhoo Cheon, Seong-Gyu Ko, Hyunsu Bae, Woojin Kim

**Affiliations:** 1 Department of Physiology, College of Korean Medicine, Kyung Hee University, Seoul 02447, Korea; tydsla123@naver.com (S.K.); mibdna@khu.ac.kr (J.-H.L.); emilly86@naver.com (H.Y.); hbae@khu.ac.kr (H.B.); 2 Cancer Preventive Material Development Research Center, College of Korean Medicine, Kyung Hee University, Seoul 02447, Korea; 3 Korean Medicine-Based Drug Repositioning Cancer Research Center, College of Korean Medicine, Kyung Hee University, Seoul 02447, Korea; hreedom@khu.ac.kr (C.C.); epiko@khu.ac.kr (S.-G.K.); 4 Department of East-West Medicine, Graduate School, Kyung Hee University, Seoul 02447, Korea; khan2296@naver.com

**Keywords:** [6]-shogaol, GABA, neuropathic pain, oxaliplatin, serotonin, *Zingiber officinale* roscoe

## Abstract

Although oxaliplatin is a well-known anti-cancer agent used for the treatment of colorectal cancer, treated patients often experience acute cold and mechanical allodynia as side effects. Unfortunately, no optimal treatment has been developed yet. In this study, [6]-shogaol (10 mg/kg, i.p.), which is one of the major bioactive components of *Zingiber officinale* roscoe (*Z. officinale*), significantly alleviated allodynia induced by oxaliplatin (6 mg/kg, i.p.) injection. Cold and mechanical allodynia were assessed by acetone drop and von Frey filament tests, respectively. The analgesic effect of [6]-shogaol was blocked by the intrathecal injection of 5-HT1A, 5-HT3, and GABAB receptor antagonists, NAN-190 (1 μg), MDL-72222 (15 μg), and CGP 55845 (10 μg), respectively. Furthermore, oxaliplatin injection lowered the GABA concentration in the superficial laminae of the spinal dorsal horn, whereas [6]-shogaol injection significantly elevated it. The GAD (glutamic acid decarboxylase) 65 concentration also increased after [6]-shogaol administration. However, pre-treatment of NAN-190 completely inhibited the increased GABA induced by [6]-shogaol in the spinal dorsal horn, whereas MDL-72222 partially blocked the effect. Altogether, these results suggest that [6]-shogaol could attenuate oxaliplatin-induced cold and mechanical allodynia through 5-HT_1A_ and 5-HT_3_ receptor antagonists located in the GABAergic neurons in the spinal dorsal horn in mice.

## 1. Introduction

Oxaliplatin is a platinum-based anti-cancer agent widely used for the treatment of metastatic (palliative) and advanced (adjuvant chemotherapy) colorectal cancer and adjuvant chemotherapy [1]. Moreover, as it does not induce nephrotoxicity or ototoxicity as first- and second-generation platinum-based chemotherapeutic drugs, it is known to be relatively safe [2]. However, oxaliplatin can also cause other side-effects such as fatigue, nausea, vomiting, and neuropathic pain [3]. Among them, peripheral neuropathic pain induced shortly after oxaliplatin injection is a serious problem that can even result in the discontinuation of treatment, thus delaying the treatment schedule of cancer patients [4]. The symptoms of oxaliplatin-induced peripheral neuropathic pain are characterized by dysesthesia and paresthesia, which refers to the presence of tingling, numbness, pressure, and abnormal cold or warm sensations [5]. Acutely, it can occur a few hours after administration and can last for up to several months when untreated [6]. Furthermore, it has been reported that continuous experiences of acute pain could cause long-lasting changes within the peripheral and central nervous systems, leading to chronic pain [7], This suggests that acute pain treatment may also be important in chemotherapy-induced neuropathic pain.

Although various treatments such as gabapentin and tricycle antidepressants (TCAs) are used to attenuate the pain, updated guidelines of the American Society of Clinical Oncology (ASCO) recommends duloxetine as the only agent to attenuate the CIPN [8]. However, duloxetine also has its own adverse effects such as nausea, dry mouth, headache, and dizziness [9]. Therefore, it is necessary to find a treatment that can effectively treat oxaliplatin-induced neuropathic pain without causing side effects.

Serotonin (5-hydroxytryptamine; 5-HT) is a monoamine neurotransmitter widely distributed in the nervous system [10]. As part of the descending pain inhibitory system it is synthesized in the rostro ventromedial medulla (RVM), and when pain occurs, it attenuates the pain by increasing 5-HT in the spinal cord [11] and 5- exerts analgesic effects depending on the site of action and the receptor subtype [12]. In the central and peripheral nervous systems 5-HT receptors are found [13]. They are divided into seven different classes of receptors that are differentiated into 15 subtypes [14], and modulate the release of other neurotransmitters such as glutamate, dopamine, norepinephrine, and gamma-Aminobutyric acid (GABA) [15]. Several studies conducted on different animal models of pain [16,17], such as formalin-induced pain, have demonstrated that by modulating the 5-HT system, neuropathic pain can be alleviated [11,18,19]. In addition, 5-HT receptors have also been known to be related to analgesia, as its receptors knocked-out resulted in pain enhancement [16,20].

GABA is a well-known inhibitory neurotransmitter of the central nervous system (CNS). The major role of spinal GABA is to inhibit neuronal excitability [21]. It is known to cause hyperpolarization by opening the Cl^-^ ion channel and inhibit the genesis of action potential [22]. Some 5-HT receptors such as 5-HT_1A_, 5-HT_3_, or 5-HT_7_ receptors are reported to be located in GABAergic neurons and induce the release of GABA [12,23]. An increased GABA concentration in the spinal cord was reported to induce an analgesic effect, whereas the downregulation of GABA or GAD (glutamic acid decarboxylase) concentration contributed to neuropathic pain [24,25]. GAD exists in two isoforms, GAD65 and GAD67, and they are both known to synthesize GABA [26]. GAD67 is distributed in neuronal cell bodies while GAD65 is present at nerve terminals. GAD65 is known to synthesize GABA neurotransmitters, whereas GAD67 has little relation to the GABA neurotransmitter [27]. In an animal model of paclitaxel-induced neuropathic pain addition, GABA application reversed the increased excitability of chemotherapeutic agent-treated rats in electrophysiological recording suggesting that GABA can be a potential therapeutic target of chemotherapy-induced peripheral neuropathy (CIPN) [28].

In our previous study, we demonstrated that *Zingiber officinale* Roscoe (*Z. officinale*) could effectively attenuate oxaliplatin-induced neuropathic pain in mice [29]. In this study, the analgesic effect of *Z. officinale* was shown to be mediated by spinal 5-HT_1A_ and 5-HT_3_ receptors, as the intrathecal treatment of both antagonists significantly blocked the analgesic effect of *Z. officinale.* However, which component of *Z. officinale* that played a major role in the analgesic effect of *Z. officinale* remains unstudied. Although *Z. officinale* is composed of various components, we focused on [6]-shogaol as it has been reported to be one of the major bioactive components in *Z. officinale* [30]. Moreover, to date, its effect on oxaliplatin- and chemotherapy-induced pain has never been studied.

Thus, in this study, we aimed to assess the analgesic effect of [6]-shogaol on oxaliplatin-induced neuropathic pain. Second, we studied the role of spinal 5-HT receptors in the analgesic effect of [6]-shogaol, and assessed whether the spinal GABA is affected by [6]-shogaol treatment. Finally, we focused on the relation of spinal 5-HT receptors and GABA concentration by observing the GABA change after 5-HT receptor antagonists treatments.

## 2. Results

### 2.1. Single Oxaliplatin Injection Induced Cold and Mechanical Allodynia in Mice

**Figure 1:**
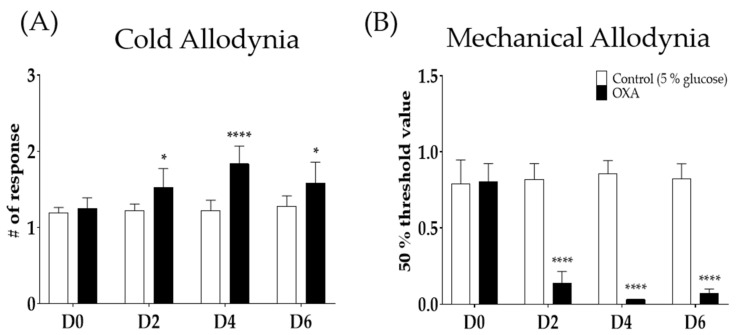
Single intraperitoneal administration of 6 mg/kg of oxaliplatin induced cold and mechanical allodynia in mice (**A**,**B**). Cold allodynia was assessed by using acetone drop test (**A**). Mechanical allodynia was measured by using von Frey filament test (**B**). Control group received 5% glucose as control. D0; day of oxaliplatin or 5% glucose injection, D2; two days following injection, D4; four days after treatment, and D6; six days after treatment. Data are presented by mean ± standard error of the mean (SEM). Control: *n* = 6, oxaliplatin: *n* = 6. * *p* < 0.05, **** *p* < 0.0001 vs. Control with two-way ANOVA followed by Sidak’s multiple comparisons test.

A single oxaliplatin (6 mg/kg, i.p.) injection increased the number of responses to acetone drop (A) and lowered the 50% threshold value to von Frey filament stimuli (B). Oxaliplatin administration induced cold and mechanical allodynia from day 2 (D2) to day 6 (D6) in mice. Responses to the acetone drop was significantly increased in the group treated with oxaliplatin compared to 5% glucose. Five percent glucose was used as a control to oxaliplatin (Figure 1A,B). The responses to mechanical stimuli were also significantly lowered from D2 to D6 after oxaliplatin injection compared to the control (Figure 1B).

### 2.2. Intraperitoneal Injection of [6]-Shogaol Decreased Cold and Mechanical Alloydnia in Mice

**Figure 2:**
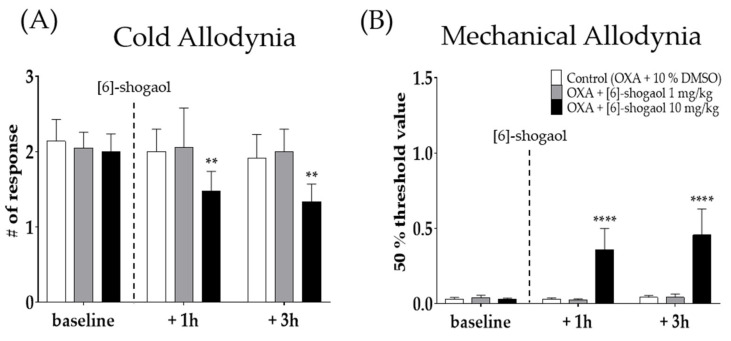
The analgesic effect of intraperitoneal injection of [6]-shogaol in oxaliplatin-induced allodynia in mice (**A**,**B**). Behavioral tests were conducted four days after 6 mg/kg of oxaliplatin (i.p.) injection. Cold allodynia (**A**) was assessed by using acetone drop test and mechanical allodynia (**B**) was measured by using von Frey filament test. Control group mice were treated with 10% of DMSO. Two different doses of [6]-shogaol was used (1 and 10 mg/kg). Baseline: before the injection of 10% DMSO or [6]-shogaol. +1 h: 1 h after 10% DMSO or [6]-shogaol treatment. +3h: 3 h after injection of 10% DMSO or [6]-shogaol administration. Data are presented by mean ± SEM. Control (OXA + 10% DMSO): *n* = 6, OXA + [6]-shogaol 1 mg/kg: *n* = 6, OXA + [6]-shogaol 10 mg/kg: *n* = 6. ** *p* < 0.01, **** *p* < 0.0001 vs. Control with two-way ANOVA followed by Tukey’s multiple comparisons test.

Two different doses of [6]-shogaol (1 and 10 mg/kg) were injected intraperitoneally four days after oxaliplatin injection, when cold and mechanical allodynia were significantly induced in mice. Behavioral tests were assessed before (baseline), 1 h, and 3 h after the injection of [6]-shogaol or 10% dimethyl sulfoxide (DMSO). Ten percent DMSO was used as a control to [6]-shogaol. Cold (Figure 2A) and mechanical (Figure 2B) allodynia was significantly decreased until 3h following 10 mg/kg of [6]-shogaol administration; however, the 1 mg/kg and 10% DMSO treated group did not show any difference. Thus, 10 mg/kg of [6]-shogaol was used in our subsequent experiments.

### 2.3. Intrathecal Serotonergic Receptor Antagonists Completely Block the Effect of [6]-Shogaol

**Figure 3:**
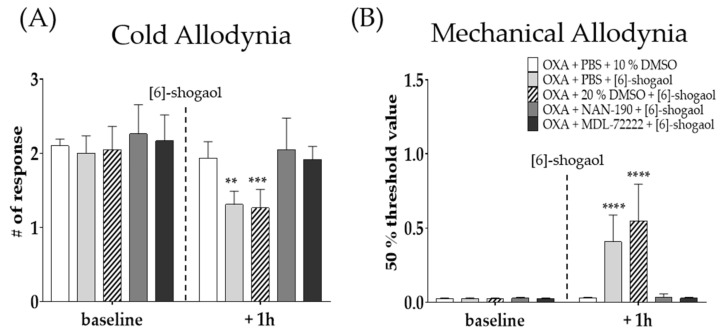
Effect of intrathecal injection of NAN-190 and MDL-72222 on the analgesic effect of [6]-shogaol on oxaliplatin-induced cold (**A**) and mechanical (**B**) allodynia. NAN-190 and MDL-72222 are 5-HT_1A_ and 5-HT_3_ receptor antagonists, respectively. Cold allodynia was assessed by using acetone drop test (**A**) and mechanical allodynia was measured by using von Frey filament test (**B**). All groups received oxaliplatin injection four days prior to behavior tests. PBS and 20% DMSO was used as control for NAN-190 and MDL-72222, respectively. Ten percent DMSO or [6]-shogaol was intraperitoneal injected 20 min after intrathecal injection of PBS, 20% DMSO, NAN-190, and MDL-72222. Baseline: before injection of PBS, 10%, and 20% DMSO, NAN-190, MDL-72222, and [6]-shogaol. + 1h: 1 h after injection of PBS, 10 and 20% DMSO, NAN-190, MDL-72222, and [6]-shogaol. Data are presented by mean ± SEM. OXA + PBS + 10% DMSO: *n* = 5, OXA + PBS + [6]-shogaol: *n* = 7, OXA + 20% DMSO + [6]-shogaol: *n* = 7, OXA + NAN-190 + [6]-shogaol: *n* = 7, OXA + MDL-72222 + [6]-shogaol: *n* = 6. ** *p* < 0.01, *** *p* < 0.001, ****, *p* < 0.0001 vs. Control with two-way ANOVA followed by Tukey’s multiple comparisons test.

To observe the role of serotonergic receptors in the analgesic effect of [6]-shogaol, two different receptor antagonists; NAN-190 (5-HT_1A_ receptor antagonist) and MDL-72222 (5-HT_3_ antagonist) were administered intrathecally 20 min before [6]-shogaol treatment. Behavioral tests were performed four days after oxaliplatin injection. Phosphate buffered saline (PBS) and 20% DMSO were used as a control to NAN-19 and MDL-72222, respectively. All drugs were administered in the lumbar 4–5 segments of spinal cord. The results show that both spinal 5-HT_1A_ and 5-HT_3_ receptor antagonists completely inhibited the analgesic effect of [6]-shogaol on oxaliplatin-induced cold (Figure 3A) and mechanical (Figure 3B) allodynia. These results suggest that the analgesic effect of [6]-shogaol is mediated by both spinal 5-HT_1A_ and 5-HT_3_ receptors present in the lumbar 4–5 spinal cord segments.

### 2.4. GABA_B_ Receptor Antagonist Prevented the Analgesic Effect of [6]-Shogaol

**Figure 4:**
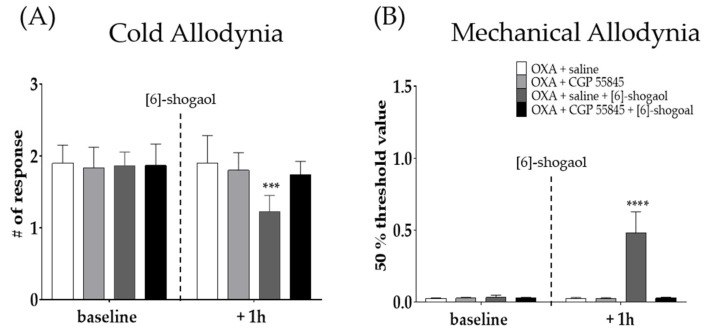
Intrathecal administration of GABA_B_ receptor antagonist prevented the analgesic effect of [6]-shogaol in oxaliplatin-induced cold (**A**) and mechanical allodynia (**B**). CGP 55845 is GABA_B_ receptor antagonist. Cold allodynia was assessed by using acetone drop test (**A**) and mechanical allodynia was measured by using von Frey filament test (**B**). All group received oxaliplatin injection four days prior to behavior test. Saline was used as control for CGP 55845. [6]-shogaol was intraperitoneal injected 20 min after intrathecal injection of saline and CGP 55845. Baseline: before injection of saline, CGP 55845, and [6]-shogaol. +One h: 1 h after injection of saline, CGP 55845, and [6]-shogaol. Data are presented by mean ± SEM. OXA + saline: *n* = 5, OXA + CGP 55845: *n* = 5, OXA + saline + [6]-shogaol: *n* = 6, OXA + CGP 55845 + [6]-shogaol: *n* = 5. *** *p* < 0.001, **** *p* < 0.0001 vs. OXA + saline with two-way ANOVA followed by Tukey’s multiple comparisons test.

To assess the involvement of spinal GABA in the analgesic effect of [6]-shogaol, CGP 55845 (GABA_B_ receptor antagonist) was intrathecally injected 20 min before [6]-shogaol treatment. GABA_B_ receptor is a metabotropic receptor largely presented on the superficial spinal dorsal horn laminae I-II and is known to be involved in inhibitory neurotransmitter systems at stimulation [31]. Saline was used as a control to CGP 55845. Intrathecal injection of CGP 55845 blocked the analgesic effect of [6]-shogaol against cold (Figure 4A) and mechanical (Figure 4B) allodynia induced by oxaliplatin. These results suggest that GABA is involved in the analgesic effect of [6]-shogaol.

### 2.5. [6]-Shogaol Increased GABA Concentration in the Superficial Laminae of the Dorsal Horn of the Mice Spinal Cord

**Figure 5:**
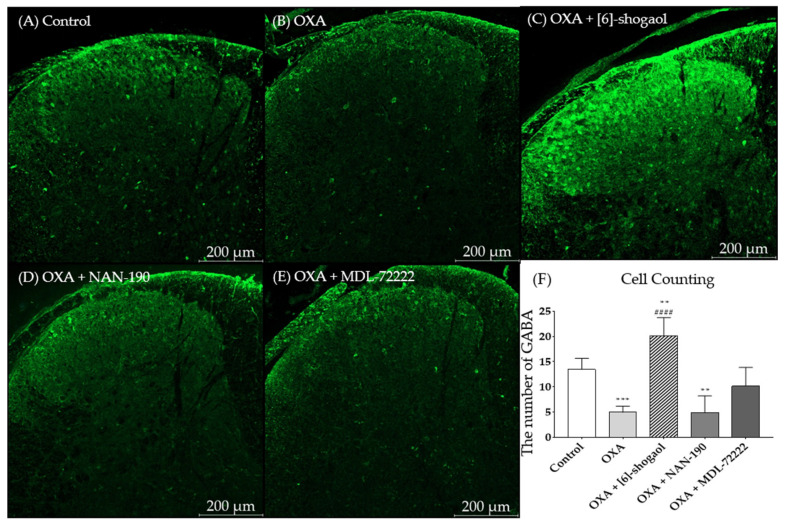
Change of GABA concentration after [6]-shogaol and 5-HT receptor antagonists administration in superficial laminae of dorsal horn of lumbar 4 -5 spinal cord segments. The lumbar 4–5 segment of the spinal cord of control, (**A**) oxaliplatin (OXA), (**B**) [6]-shogaol, (**C**) OXA + NAN-190, (**D**) OXA + MDL-72222, and (**E**) treated mice were stained with GABA antibodies. After immunohistochemistry GABA (green) was quantified (**F**). All groups, except control group, received oxaliplatin injection four days prior to the experiments. Control group received 5% glucose. Data are presented by mean ± SEM. Control: *n* = 5, OXA: *n* = 6, [6]-shogaol: *n* = 6, NAN-190: *n* = 5, MDL-72222: *n* = 5. ** *p* < 0.01, *** *p* < 0.001 vs. Control and #### *p* < 0.0001 vs. OXA with one-way ANOVA followed by Tukey’s multiple comparisons test.

**Figure 6:**
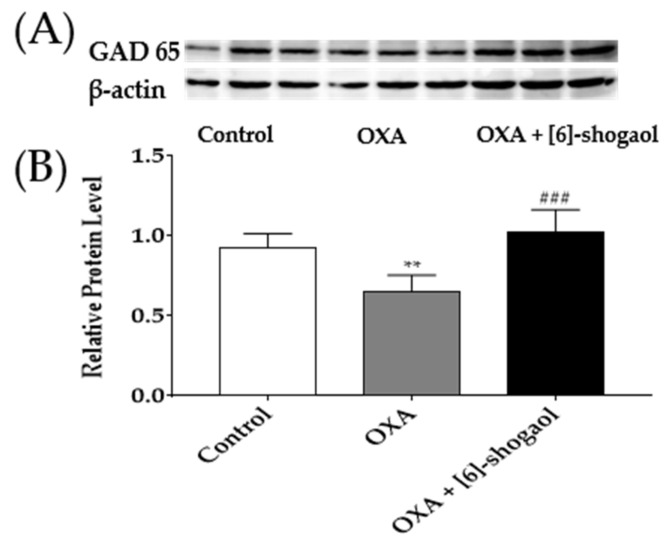
GAD65 protein concentration in the lumbar 4–5 spinal cord segments following intraperitoneal injection of [6]-shogaol. GAD65 protein binding band density image (**A**). The density of GAD65 protein concentration was quantified compared to β-actin (**B**). Control group received 5% glucose. OXA and OXA + [6]-shogaol groups received oxaliplatin injection four days prior to experiments. Data are presented by mean ± SEM. Control: *n* = 6, OXA: *n* = 6, OXA + [6]-shogaol: *n* = 6. ** *p* < 0.01 vs. Control and ### *p* < 0.0001 vs. OXA with one-way ANOVA followed by Tukey’s multiple comparisons test.

To explore the interaction between the serotonergic receptors and the GABA concentration in the spinal cord, the change in the GABA concentration was assessed through immunohistochemistry. The experiment was performed in the superficial dorsal horn of the lumbar 4–5 spinal cord segments in mice. After oxaliplatin injection the GABA concentration was downregulated in the spinal dorsal horn (Figure 5B); whereas it was significantly upregulated after 10 mg/kg of [6]-shogaol injection (Figure 6C, *p* < 0.01 vs control, *p* < 0.0001 vs oxaliplatin). Furthermore, this effect of [6]-shogaol was completely blocked by NAN-190 (5-HT_1A_ receptor antagonist) (Figure 5D). However, MDL-72222 (5-HT_3_ receptor antagonist) only partially inhibited the effect of [6]-shogaol (Figure 5E) as it did not show any significant differences compared to the control, oxaliplatin-, and [6]-shogaol-treated groups (Figure 6F). The quantification of GABA was conducted by counting the strong intensity and clear shape of GABA (green) in the superficial dorsal horn. Altogether, these results show that [6]-shogaol can increase the GABA concentration in the spinal dorsal horn and that this effect is mediated by 5-HT_1A_ and 5-HT_3_ (partially) receptors located in the superficial laminae of the spinal dorsal horn.

### 2.6. Administration of [6]-Shogaol Increased GAD65 Protein in the Spinal Cord in Mice

To further confirm the activation of GABA after [6]-shogaol injection, changes of GAD65 protein concentration in the lumbar 4–5 spinal cord segments were determined by western blot analysis. The concentration of GAD65 protein was significantly reduced following the oxaliplatin injection compared to the 5% glucose (control). However, the intraperitoneal administration of [6]-shogaol reversed the decreased GAD65 protein of the oxaliplatin treatment (OXA + [6]-shogaol). These results demonstrate that [6]-shogaol can increase GABA concentration by increasing the GAD65 concentration in the spinal cord.

## 3. Discussion

In this study, we have demonstrated that the intraperitoneal injection of [6]-shogaol could significantly attenuate oxaliplatin-induced cold and mechanical allodynia in mice. Two different doses (i.e., 1 and 10 mg/kg) of [6]-shogaol were administered and only 10 mg/kg of [6]-shogaol showed an analgesic effect that lasted to 3h following treatment. Moreover, as the underlying mechanism of action, we showed that both spinal 5-HT_1A_ and 5-HT_3_ receptors are involved as the intrathecal administration of NAN-190 (5-HT_1A_ receptor antagonist) and MDL-72222 (5-HT_3_ receptor antagonist) prevented the analgesic effect of [6]-shogaol. Finally, we have shown that the administration of [6]-shogaol could significantly increase GABA concentration in the superficial laminae of the dorsal horn of the lumbar 4–5 spinal cord segments, and that spinal GABA_B_ receptors are also involved in the effect of [6]-shogaol. To our knowledge this is the first study to show the analgesic effect of [6]-shogaol in oxaliplatin-induced pain and to report the relation of [6]-shogaol and spinal GABA concentration.

Oxaliplatin-induced neuropathic pain may even occur after a few hours of administration and can interrupt the chemotherapy schedules in cancer patients [4]. In this study, 6 mg/kg of oxaliplatin was used to mimic the allodynia and hyperalgesia that occurs after administration in humans. In the clinic, oxaliplatin is known to be used at 85 mg/m^2^ every two weeks or 130 mg/m^2^ every three weeks, with the maximum tolerated dose of 200 mg/m^2^. These concentrations correspond approximately to 6 mg/kg used in this study [32]. Various pathways of oxaliplatin-induced neuropathic pain has been reported, such as mitochondrial damage [33], nuclear DNA damage [34], glia activation [35], and ion channels dysfunction [36,37]; however its mechanism of action is still not completely understood.

For many years, our lab has put efforts into understand the pathophysiological mechanism of oxaliplatin-induced neuropathic pain and to find an optimal treatment method through experiments. Among them, modulating the 5-HT system, especially its receptors located in the spinal cord, appeared to be a good target, as intrathecally administered receptor agonists succeeded to alleviated the pain induced by chemotherapy [29] Furthermore, in our previous paper, *Z. officinale* attenuated cold and mechanical allodynia induced by oxaliplatin via the action of spinal 5-HT_1A_ receptors [29]. In this study, 10 mg/kg of [6]-shogaol, which is a component of *Z. officinale*, significantly alleviated both cold and mechanical allodynia induced by oxaliplatin for 3h. According to a study, the LD50 of [6]-shogaol was reported to be 109.2 mg/kg when injected intraperitoneally in mice [38]. Furthermore, a clinical study conducted in humans has reported that the maximum recommended dose for dried ginger extract is 2.5 g/day. As the dried ginger extract contains approximately 1% to 4% of shogaols [39], the dose used in our experiments (i.e., 1 and 10 mg/kg) should cause no serious side effects in treated mice.

[6]-Shogaol is one of the major bioactive components of *Z. officinale* that has been widely used as a herbal medicine for the prevention and treatment of various diseases [30,40]. In addition, it was found that [6]-shogoal could passively cross the blood–brain barriers (BBB) suggesting that [6]-shogaol could act directly on the CNS [41]. From other research groups, [6]-shogaol was reported to have an analgesic effect as it decreased streptozotocin-induced diabetic neuropathic pain in rodents [42]; however, its effect has never been studied on CIPN.

In this study, the effect of [6]-shogaol was mediated by spinal 5-HT_1A_ and 5-HT_3_ receptors, as both receptor antagonists (NAN-190 and MDL-72222, respectively) completely inhibited the effect of [6]-shogaol. NAN-190 and MDL-72222 are reported to have a high binding affinity for 5-HT_1A_ and 5-HT_3_ receptors, respectively [43,44]. In addition, the intrathecal injection of the two antagonists effectively blocks each receptor [45,46]. In our previous study, the analgesic effect of *Z. officinale* was also mediated by spinal 5-HT_1A_ and 5-HT_3_ receptors [29]. Intrathecal administration with 5-HT_1A_ receptor antagonist (NAN-190) blocked the analgesic effect of *Z. officinale* against both cold and mechanical allodynia, whereas 5-HT_3_ receptor antagonist (MDL-72222) only blocked the analgesic effect against cold allodynia. In this study, both antagonists significantly inhibited the analgesic effect of [6]-shogaol. However, although the 5-HT_1A_ receptor antagonist also blocked the [6]-shogaol-induced activation of GABA concentration in the spinal cord, the 5-HT_3_ receptor antagonist has failed to completely block the effect suggesting that the 5-HT_3_ receptor may involve other pathways than GABA in mediating the analgesic effect of [6]-shogaol.

The 5-HT_1A_ receptor is a G-protein-coupled receptor (GPCR), located in the dorsal and median raphe nuclei, cortical, and limbic areas of the brain and are distributed according to the rostro-caudal gradient within the spinal cord [47]. In a lumbar 5–6 spinal nerve ligation-induced neuropathic pain, the intrathecal administration of 5-HT reversed tactile allodynia [48]. Furthermore, in a formalin-induced animal model of pain, the intrathecal injection of 5-HT_1A_ agonist (8-OH-DPAT) induced analgesia [12]. These results suggest that by activating the 5-HT_1A_ receptors at the spinal cord level the pain could be attenuated.

The 5-HT_3_ receptors are ligand-gated ion channels (LGICs) and differ from all other 5-HT receptors structurally and functionally [49]. GPCR activates an intracellular second messenger cascade to generate an excitatory or inhibitory response, whereas LGICs produce an excitatory or inhibitory response through the depolarization of plasma membranes [50]. However, they are both expressed in the superficial laminae of the spinal dorsal horn [51]. Several studies have also demonstrated the relation of the 5-HT_3_ receptor in pain alleviation. Intrathecal administration of the 5-HT-induced analgesic effect on the tail-flick and the hot-plate test was reported to be mediated by 5-HT_3_ receptors [52]. Additionally, the intrathecal injection of the 5-HT_3_ receptor agonist, 2-methylserotonin, produced dose-dependent antinociception [53].

In our study, 10 mg/kg of [6]-shogaol significantly increased GABA concentration in the spinal cord. Moreover, this increase was completely inhibited by 5-HT_1A_ receptor antagonist (NAN-190) and partially blocked by 5-HT_3_ receptor antagonist (MDL-72222), suggesting a close relation of spinal 5-HT receptors and GABA in the analgesic effect of [6]-shogaol. GABA is known to inhibit the excitability of neurons as an inhibitory neurotransmitter in the CNS [21]. It is synthesized via the GAD and pyridoxal phosphate from glutamate [54]. There are reports that the decreased synthesis of GABA, the down-concentration of GAD, and the disturbed function of GABA receptors, contributes to neuropathic pain [55]. In an animal model of CIPN, the GABA concentration decreased after paclitaxel injection, which is a taxane-based chemotherapeutic agent, suggesting the involvement of GABA in chemotherapy-induced neuropathic pain [28]. Furthermore, in an animal model of oxaliplatin-induced neuropathic pain, the GABA was also shown to decrease on the periaqueductal gray [56]; however, its change in the spinal cord is not clearly understood yet. In our study, the spinal GABA concentration significantly decreased after oxaliplatin injection. In addition, the analgesic effect of [6]-shogaol was blocked by GABA_B_ receptor antagonist, demonstrating the involvement of spinal GABA in oxaliplatin-induced pain. The GABA receptor exists of three subtypes: A, B, and C. Among them, GABA_A_ and GABA_B_ receptor are expressed throughout the spinal gray matter, and are concentrated in the spinal dorsal horn laminae I-II. Both receptors are well known to be involved in pain control. Especially, it has been well demonstrated that GABA_B_ receptors regulate pain transfer [31,57,58]. In addition, GABA_B_ receptor stimulation directly reduces neurotransmitter release, which regulates the activity of various excitatory and inhibitory neurotransmitter systems [31].

Through the reverse transcription-polymerase chain reaction, it was found that both 5-HT_1A_ and 5-HT_3_ receptors are collocated in the GABAergic interneurons in the spinal cord [59,60]. About half of the GABAergic neurons located in the spinal dorsal horn are known to express 5-HT_1A_ receptors [61]. In addition, the analgesic effect of 5-HT_3_ receptor agonist administered intrathecally was blocked by both GABA and 5-HT_3_ receptor antagonist, suggesting that GABA is involved in the effect induced by spinal 5-HT_3_ receptor activation [53]. However, the relation of spinal 5-HT receptors and GABA concentration is not fully understood, and well-designed research is needed. To our knowledge, this is the first study to show that intraperitoneal [6]-shogaol injection could increase GABA concentration in the spinal cord, and that in the increase of GABA concentration, 5-HT receptors are involved.

## 4. Materials and Methods

### 4.1. Animals

Adult C57BL/6 mice (6 weeks old) were obtained from Daehan biolink (Chungbuk, Korea). They were housed in a specific pathogen-free animal center where the temperature and humidity were maintained at 23 ± 2 °C and 65 ± 5%, respectively. A 12 h light/dark cycle was fixed, and food and water were provided ad libitum. All experimental protocols were approved by the Kyung Hee University Animal Care and Use Committee (KHUASP(SE)-20-448) on 15 November and were conducted in accordance with the guidelines of the International Association for the Study of Pain [62].

### 4.2. Oxaliplatin-Induced Neuropathic Pain

Oxaliplatin (Sigma Aldrich, St. Louis, MO, USA) was dissolved in a 5% glucose solution at a concentration of 2 mg/mL, as in our previous study [29]. Oxaliplatin was administered intraperitoneally at a dose of 6 mg/kg to mice. The control group was injected with the same amount of 5% glucose. To assess whether oxaliplatin administration could significantly induce cold and mechanical allodynia in mice, behavioral tests were conducted before (baseline), two (D2), four (D4), and six (D6) days after its injection (Figure 1A,B). All experiments were conducted on day four when cold and mechanical allodynia were strongly induced compared to control.

### 4.3. Adminsitration of [6]-Shogaol

[6]-Shogaol (FUJIFILM Wako Pure Chemical Corporation, Osaka, Japan) was dissolved in a 10% dimethyl sulfoxide (DMSO) solution at a concentration of 0.15 and 1.5 mg/mL. [6]-Shogaol was intraperitoneally injected at a dose of 1 and 10 mg/kg to mice [42]. For control, the same volume (0.2 mL) of 10% DMSO was injected. [6]-Shogaol or 10% DMSO was administered four days (D4) after oxaliplatin injection (Figure 2A,B).

### 4.4. Intrathecal Injection of 5-HT and GABA_B_ Receptor Antagonists

To clearly observe the role of spinal 5-HT receptors in the analgesic effect of [6]-shogaol, NAN-190 (5-HT_1A_ receptor antagonist, 1 μg, concentration 0.2 μg/μL), MDL-72222 (5-HT_3_ receptor antagonist, 15 μg, concentration 3 μg/μL), and CGP 55845 (GABA_B_ receptor antagonist, 10 μg) were intrathecally injected 20 min before the administration of [6]-shogaol. NAN-190, MDL-72222, and CGP 55845 were dissolved in phosphate buffered saline (PBS), 20% DMSO, and saline, respectively. Control group mice received solvents (PBS, 20% DMSO, or saline). Behavioral assessments were conducted twice, before the injection of 5-HT receptor antagonists and 1h after [6]-shogaol treatment. Both 5-HT and GABA_B_ receptor antagonists were purchased from Tocris (Cookson, UK) and Sigma (St. Louis, MO, USA). Intrathecal injection of antagonist solutions (5 μL) were performed at the lumbar 4–5 intervertebral level by using a Hamilton syringe (Hamilton Company, Reno, NV, USA) after isoflurane anesthesia [63].

### 4.5. Behavioral Assessments

To assess behavioral changes in mice, acetone drop and von Frey filament tests were conducted to measure cold and mechanical allodynia, respectively [29]. All animals were placed on a metal mesh floor and were caged in an inverted clear plastic cage (12 × 8 × 6 cm^3^) for 30 min before all measurements for acclimation. To measure responses to cold stimuli, acetone drop (10 μL) was applied on each mid-plantar hind paw of mice. Acetone drop was applied three times on both paws and licking and shaking in responses to acetone drop were observed for 30 s. The ‘# of responses’ in the Y-axis (Figure 1A) refers to the average number of responses to six times of assays (Figure 1A).

To measure responses to mechanical stimuli, a series of von Frey filaments (bending force of 0.02, 0.04, 0.07, 0.16, 0.4, 0.6, 1, 1.4, and 2 g, Stoelting, Kiel, WI, USA) were applied on the mid-plantar hind paws. Using the Dixon’s up–down method and Chaplan’s calculation method obtained average from both hind paws [64,65]. The “50% threshold value” in the Y-axis refers to the average number of responses to both hind paws.

### 4.6. Immunohistochemistry (IHC)

Immunohistochemistry was conducted as in our previous studies [66]. In brief, four days after oxaliplatin injection when pain was significantly induced, mice were anesthetized with isoflurane and transcardially perfused with 0.1 M PBS and fixed with a freshly prepared solution consisting of 4% paraformaldehyde (PFA) in 0.1 M phosphate buffer (pH 7.4). The lumbar 4–5 spinal cord segments were collected in fixed mice, and were post-fixed overnight in 4% PFA and transferred into 30% sucrose. Samples of fixed lumbar segment were embedded in optimal cutting temperature (OCT) compound (Sakura Finetek, Tokyo, Japan) and kept in a box on −80 °C. Samples of lumbar spinal cord segments were sectioned at 20 μm thickness using cryostat (Microm HM 505N; Thermo Fisher Scientific, Waltham, MA, USA). These sections were collected mounted onto glass slides (Matsunami, Osaka, Japan), and air-dried overnight. After rinsing the glass slide with PBS for 3 times incubated in 0.2% Triton X-100 in 0.5% bovine serum albumin (BSA; BOVOGEN biologics, East Keilor, Australia) solution at room temperature for 45 min for permeability. After rinsing the glass slide with PBS, it was blocked with 3% BSA in 0.05% PBST for 30min. After PBS rinses, these sections were incubated overnight with the primary antibody: rabbit anti-GABA (Sigma, A2052, 1: 1000) in 0.1% BSA. After rinses, the secondary antibody, applied at room temperature for 2h, was anti-rabbit-immunoglobulin G (IgG) labeled with Alexa Fluor 488 (Invitrogen, Carlsbad, CA, USA, 1:1000) in 0.1% BSA. Sections were washed in 0.1 M PBS and mounted in Vectashield mounting medium with DAPI (Vector Labs, Burlingame, CA) and immunohistochemical images were obtained by using Confocal laser microscope (LSM 5 Pascal, Zeiss, Oberkochen, Germany). GABA-positive cells were observed in both sides of the spinal dorsal horn laminae I and II. To determine GABA positive cells image J plug in cell counter was used and average were obtained.

### 4.7. Western Blots

Lumbar 4–5 spinal cord segments were collected as mentioned above. Tissue samples were homogenized with radioimmunoprecipitation (RIPA) buffer (Thermo Fisher Scientific, MA, USA) plus phosphatase inhibitor cocktail (100×, Thermo Fisher Scientific, MA, USA). After homogenization, tissues were incubated for 15 min on ice and centrifuged at 13,000× *g* at 4 °C for 20 min. The supernatant was assayed using the Bradford protein assay (BIO-RAD, CA, USA). Thirty micrograms of protein samples were loaded and run on a 8% Tris-glycine sodium dodecyl sulfate-polyacrylamide gel followed by electrophoresis, then were transferred to a nitrocellulose membrane (BIO-RAD, CA, USA). The membranes were blocked with 5% skim milk in 0.05% Tris Buffered Saline with Tween 20 (TBS-T). After blocking, incubated with primary antibody for overnight at 4 °C with rabbit anti-GAD65 (Cell Signaling Technology, MA, USA, 1:1000) in 5% skim milk. After rinsing the membrane with TBS-T, it was incubated with secondary antibody for 2 h at room temperature with Goat anti-rabbit Ig G/ HRP antibody (Solarbio, Beijing, China, 1:2000) in 5% skim milk. Bands were detected using enhanced chemiluminescence (ECL) solution (Donginbio, Seoul, Korea, A:B = 1:1) and imaged with davinch. Density image was quantified by using image J. GAD65 bands were normalized using the amount of β-actin.

### 4.8. Statistical Analysis

All data were presented as mean ± standard error of the mean (SEM). Statistical analysis and graphic works were performed by using Prism 7.0 (GraphPad software, La Jolla, CA, USA). Two-way ANOVA (analysis of variance) followed by Sidak’s or Tukey’s post-tests for multiple comparisons and Student’s t-tests were used for statistical analyses. In all cases, *p* < 0.05 was considered to indicate significant differences.

## 5. Conclusions

Our study demonstrated that the intraperitoneal injection of [6]-shogaol could significantly attenuate oxaliplatin-induced neuropathic pain through both spinal 5-HT_1A_ and 5-HT_3_ receptors. In addition, we showed that the administration of [6]-shogaol could increase the GABA and GAD65 concentration in the superficial dor-sal horn of lumbar 4–5 segments of spinal cord. Finally, this increase in GABA was shown to be mediated through spinal 5-HT_1A_ and 5-HT_3_ receptors, as their antagonists pre-treatment completely (5-HT_1A_ receptors) and partially (5-HT_3_ receptors) blocked the increased GABA concentration induced by 10 mg/kg of [6]-shogaol. Altogether, these results suggest that [6]-shogaol could be considered as an agent to treat acute al-lodynia induced by oxaliplatin treatment. Further studies are needed to fully divulge the relation of spinal 5-HT receptors and GABA concentration in pain alleviation.

## Data Availability

Data is contained within the article and supplementary material.

## References

[1] Graham M.A., Lockwood G.F., Greenslade D., Brienza S., Bayssas M., Gamelin E. (2000). Clinical pharmacokinetics of oxaliplatin: A critical review. Clin. Cancer Res. Off. J. Am. Assoc. Cancer Res..

[2] Pasetto L.M., D’Andrea M.R., Rossi E., Monfardini S. (2006). Oxaliplatin-related neurotoxicity: How and why?. Crit. Rev. Oncol. Hematol..

[3] André T., Boni C., Navarro M., Tabernero J., Hickish T., Topham C., Bonetti A., Clingan P., Bridgewater J., Rivera F. (2009). Improved overall survival with oxaliplatin, fluorouracil, and leucovorin as adjuvant treatment in stage II or III colon cancer in the MOSAIC trial. J. Clin. Oncol. Off. J. Am. Soc. Clin. Oncol..

[4] Ta L.E., Low P.A., Windebank A.J. (2009). Mice with cisplatin and oxaliplatin-induced painful neuropathy develop distinct early responses to thermal stimuli. Mol. Pain.

[5] Gebremedhn E.G., Shortland P.J., Mahns D.A. (2018). The incidence of acute oxaliplatin-induced neuropathy and its impact on treatment in the first cycle: A systematic review. BMC Cancer.

[6] Lehky T., Leonard G., Wilson R., Grem J., Floeter M. (2004). Oxaliplatin-induced neurotoxicity: Acute hyperexcitability and chronic neuropathy. Muscle Nerve.

[7] Voscopoulos C., Lema M. (2010). When does acute pain become chronic?. Br. J. Anaesth..

[8] Loprinzi C.L., Lacchetti C., Bleeker J., Cavaletti G., Chauhan C., Hertz D.L., Kelley M.R., Lavino A., Lustberg M.B., Paice J.A. (2020). Prevention and Management of Chemotherapy-Induced Peripheral Neuropathy in Survivors of Adult Cancers: ASCO Guideline Update. J. Clin. Oncol..

[9] Perahia D.G., Kajdasz D.K., Walker D.J., Raskin J., Tylee A. (2006). Duloxetine 60 mg once daily in the treatment of milder major depressive disorder. Int. J. Clin. Pract..

[10] Sommer C. (2004). Serotonin in pain and analgesia. Mol. Neurobiol..

[11] Dogrul A., Ossipov M.H., Porreca F. (2009). Differential mediation of descending pain facilitation and inhibition by spinal 5HT-3 and 5HT-7 receptors. Brain Res..

[12] Bardin L. (2011). The complex role of serotonin and 5-HT receptors in chronic pain. Behav. Pharmacol..

[13] Hoyer D., Clarke D.E., Fozard J.R., Hartig P.R., Martin G.R., Mylecharane E.J., Saxena P.R., Humphrey P.P. (1994). International Union of Pharmacology classification of receptors for 5-hydroxytryptamine (Serotonin). Pharmacol. Rev..

[14] Qi Y.X., Xia R.Y., Wu Y.S., Stanley D., Huang J., Ye G.Y. (2014). Larvae of the small white butterfly, Pieris rapae, express a novel serotonin receptor. J. Neurochem..

[15] Nichols D.E., Nichols C.D. (2008). Serotonin receptors. Chem. Rev..

[16] Ali Z., Wu G., Kozlov A., Barasi S. (1994). The actions of 5-HT1 agonists and antagonists on nociceptive processing in the rat spinal cord: Results from behavioural and electrophysiological studies. Brain Res..

[17] Cortes-Altamirano J.L., Olmos-Hernandez A., Jaime H.B., Carrillo-Mora P., Bandala C., Reyes-Long S., Alfaro-Rodríguez A. (2018). Review: 5-HT1, 5-HT2, 5-HT3 and 5-HT7 Receptors and their Role in the Modulation of Pain Response in the Central Nervous System. Curr. Neuropharmacol..

[18] Song Z., Meyerson B.A., Linderoth B. (2011). Spinal 5-HT receptors that contribute to the pain-relieving effects of spinal cord stimulation in a rat model of neuropathy. Pain.

[19] Hu B., Doods H., Treede R.D., Ceci A. (2016). Duloxetine and 8-OH-DPAT, but not fluoxetine, reduce depression-like behaviour in an animal model of chronic neuropathic pain. Neurosci. Lett..

[20] Kayser V., Elfassi I.E., Aubel B., Melfort M., Julius D., Gingrich J.A., Hamon M., Bourgoin S. (2007). Mechanical, thermal and formalin-induced nociception is differentially altered in 5-HT1A-/-, 5-HT1B-/-, 5-HT2A-/-, 5-HT3A-/- and 5-HTT-/- knock-out male mice. Pain.

[21] Keller A.F., Coull J.A., Chery N., Poisbeau P., De Koninck Y. (2001). Region-specific developmental specialization of GABA-glycine cosynapses in laminas I-II of the rat spinal dorsal horn. J. Neurosci. Off. J. Soc. Neurosci..

[22] Li K., Xu E. (2008). The role and the mechanism of gamma-aminobutyric acid during central nervous system development. Neurosci. Bull..

[23] Megumu Y., Hidemasa F. (2006). Mechanisms for the anti-nociceptive actions of the descending noradrenergic and serotonergic systems in the spinal cord. J. Pharmacol. Sci..

[24] Meisner J.G., Marsh A.D., Marsh D.R. (2010). Loss of GABAergic interneurons in laminae I-III of the spinal cord dorsal horn contributes to reduced GABAergic tone and neuropathic pain after spinal cord injury. J. Neurotrauma.

[25] Gwak Y.S., Hulsebosch C.E. (2011). GABA and central neuropathic pain following spinal cord injury. Neuropharmacology.

[26] Soghomonian J.J., Martin D.L. (1998). Two isoforms of glutamate decarboxylase: Why?. Trends Pharmacol. Sci..

[27] Pinal C.S., Tobin A. (1998). Uniqueness and redundancy in GABA production. Perspect. Dev. Neurobiol..

[28] Nashawi H., Masocha W., Edafiogho I.O., Kombian S.B. (2016). Paclitaxel Causes Electrophysiological Changes in the Anterior Cingulate Cortex via Modulation of the γ-Aminobutyric Acid-ergic System. Med. Princ. Pract. Int. J. Kuwait Univ. Health Sci. Cent..

[29] Lee J.H., Min D., Lee D., Kim W. (2021). Zingiber officinale roscoe rhizomes attenuate oxaliplatin-induced neuropathic pain in mice. Molecules.

[30] Liu Y., Liu J., Zhang Y. (2019). Research progress on chemical constituents of Zingiber officinale Roscoe. Bio. Med. Res. Int..

[31] McCarson K.E., Enna S. (2014). GABA pharmacology: The search for analgesics. Neurochem. Res..

[32] Ling B., Coudoré-Civiale M.A., Balayssac D., Eschalier A., Coudoré F., Authier N. (2007). Behavioral and immunohistological assessment of painful neuropathy induced by a single oxaliplatin injection in the rat. Toxicology.

[33] Bobylev I., Joshi A.R., Barham M., Neiss W.F., Lehmann H.C. (2018). Depletion of mitofusin-2 causes mitochondrial damage in cisplatin-induced neuropathy. Mol. Neurobiol..

[34] Avan A., Postma T.J., Ceresa C., Avan A., Cavaletti G., Giovannetti E., Peters G.J. (2015). Platinum-induced neurotoxicity and preventive strategies: Past, present, and future. Oncologist.

[35] Mannelli L.D.C., Pacini A., Micheli L., Tani A., Zanardelli M., Ghelardini C. (2014). Glial role in oxaliplatin-induced neuropathic pain. Exp. Neurol..

[36] Webster R.G., Brain K.L., Wilson R.H., Grem J.L., Vincent A. (2005). Oxaliplatin induces hyperexcitability at motor and autonomic neuromuscular junctions through effects on voltage-gated sodium channels. Br. J. Pharmacol..

[37] Siau C., Bennett G.J. (2006). Dysregulation of cellular calcium homeostasis in chemotherapy-evoked painful peripheral neuropathy. Anesth. Analg..

[38] Marmitt D.J., Shahrajabian M.H. (2021). Plant species used in Brazil and Asia regions with toxic properties. Phytother. Res..

[39] Ooi S.L., Campbell R., Pak S.C., Golombick T., Manoharan A., Ramakrishna R., Badmaev V., Schloss J. (2021). Is 6-Shogaol an Effective Phytochemical for Patients With Lower-risk Myelodysplastic Syndrome? A Narrative Review. Integr. Cancer Ther..

[40] White B. (2007). Ginger: An overview. Am. Fam. Physician.

[41] Simon A., Darcsi A., Kéry Á., Riethmüller E. (2020). Blood-brain barrier permeability study of ginger constituents. J. Pharm. Biomed. Anal..

[42] Fajrin F.A., Nugroho A.E., Nurrochmad A., Susilowati R. (2020). Ginger extract and its compound, 6-shogaol, attenuates painful diabetic neuropathy in mice via reducing TRPV1 and NMDAR2B expressions in the spinal cord. J. Ethnopharmacol..

[43] Rydelek-Fitzgerald L., Tietler M., Fletcher P.W., Ismaiel A.M., Glennon R.A. (1990). NAN-190: Agonist and antagonist interactions with brain 5-HT1A receptors. Brain Res..

[44] Fozard J. (1984). MDL 72222: A potent and highly selective antagonist at neuronal 5-hydroxytryptamine receptors. Naunyn-Schmiedeberg’s Arch. Pharmacol..

[45] Skyba D., Radhakrishnan R., Rohlwing J., Wright A., Sluka K. (2003). Joint manipulation reduces hyperalgesia by activation of monoamine receptors but not opioid or GABA receptors in the spinal cord. Pain.

[46] Radhakrishnan R., King E.W., Dickman J.K., Herold C.A., Johnston N.F., Spurgin M.L., Sluka K.A. (2003). Spinal 5-HT2 and 5-HT3 receptors mediate low, but not high, frequency TENS-induced antihyperalgesia in rats. Pain.

[47] Marlier L., Teilhac J.R., Cerruti C., Privat A. (1991). Autoradiographic mapping of 5-HT1, 5-HT1A, 5-HT1B and 5-HT2 receptors in the rat spinal cord. Brain Res..

[48] Avila-Rojas S.H., Velazquez-Lagunas I., Salinas-Abarca A.B., Barragan-Iglesias P., Pineda-Farias J.B., Granados-Soto V. (2015). Role of spinal 5-HT5A, and 5-HT1A/1B/1D, receptors in neuropathic pain induced by spinal nerve ligation in rats. Brain Res..

[49] Barnes N.M., Hales T.G., Lummis S.C., Peters J.A. (2009). The 5-HT3 receptor–the relationship between structure and function. Neuropharmacology.

[50] Brady S. (2005). Basic Neurochemistry: Molecular, Cellular and Medical Aspects.

[51] Laporte A., Koscielniak T., Ponchant M., Verge D., Hamon M., Gozlan H. (1992). Quantitative autoradiographic mapping of 5-HT3 receptors in the rat CNS using [125I] iodo-zacopride and [3H] zacopride as radioligands. Synapse.

[52] Glaum S.R., Proudfit H.K., Anderson E.G. (1988). Reversal of the antinociceptive effects of intrathecally administered serotonin in the rat by a selective 5-HT3 receptor antagonist. Neurosci. Lett..

[53] Alhaider A.A., Lei S.Z., Wilcox G.L. (1991). Spinal 5-HT3 receptor-mediated antinociception: Possible release of GABA. J. Neurosci..

[54] Petroff O.A. (2002). Book review: GABA and glutamate in the human brain. Neuroscientist.

[55] Li C., Lei Y., Tian Y., Xu S., Shen X., Wu H., Bao S., Wang F. (2019). The etiological contribution of GABAergic plasticity to the pathogenesis of neuropathic pain. Mol. Pain.

[56] Xu D., Zhao H., Gao H., Zhao H., Liu D., Li J. (2018). Participation of pro-inflammatory cytokines in neuropathic pain evoked by chemotherapeutic oxaliplatin via central GABAergic pathway. Mol. Pain.

[57] Malcangio M. (2018). GABAB receptors and pain. Neuropharmacology.

[58] Goudet C., Magnaghi V., Landry M., Nagy F., Gereau IV R.W., Pin J.-P. (2009). Metabotropic receptors for glutamate and GABA in pain. Brain Res. Rev..

[59] Wang Y.-Y., Wei Y.-Y., Huang J., Wang W., Tamamaki N., Li Y.-Q., Wu S.-X. (2009). Expression patterns of 5-HT receptor subtypes 1A and 2A on GABAergic neurons within the spinal dorsal horn of GAD67-GFP knock-in mice. J. Chem. Neuroanat..

[60] Huang J., Wang Y.-Y., Wang W., Li Y.-Q., Tamamaki N., Wu S.-X. (2008). 5-HT3A receptor subunit is expressed in a subpopulation of GABAergic and enkephalinergic neurons in the mouse dorsal spinal cord. Neurosci. Lett..

[61] Zhang Y.-Q., Gao X., Ji G.-C., Huang Y.-L., Wu G.-C., Zhao Z.-Q. (2002). Expression of 5-HT1A receptor mRNA in rat lumbar spinal dorsal horn neurons after peripheral inflammation. Pain.

[62] Zimmermann M. (1983). Ethical guidelines for investigations of experimental pain in conscious animals. Pain.

[63] Li D., Lee J.H. (2019). The Analgesic Effect of Venlafaxine and Its Mechanism on Oxaliplatin-Induced Neuropathic Pain in Mice. Int. J. Mol. Sci..

[64] Dixon W.J. (1980). Efficient analysis of experimental observations. Annu. Rev. Pharmacol. Toxicol..

[65] Chaplan S.R., Bach F.W., Pogrel J.W., Chung J.M., Yaksh T.L. (1994). Quantitative assessment of tactile allodynia in the rat paw. J. Neurosci. Methods.

[66] Kim C., Lee J.H., Kim W., Li D., Kim Y., Lee K., Kim S.K. (2016). The Suppressive Effects of Cinnamomi Cortex and Its Phytocompound Coumarin on Oxaliplatin-Induced Neuropathic Cold Allodynia in Rats. Molecules.

